# Comparing the Attitude toward the COVID-19 and the 2020/21 and 2019/20 Flu Vaccination Campaigns among Italian Healthcare Workers

**DOI:** 10.3390/vaccines9111312

**Published:** 2021-11-11

**Authors:** Giulia Collatuzzo, Riccardo Melloni, Chiara Zanotti, Giulio de Simone, Danila Pilastro, Vittorio Lodi, Paolo Boffetta

**Affiliations:** 1 Department of Medical and Surgical Sciences, University of Bologna, 40138 Bologna, Italy; giulia.collatuzzo@studio.unibo.it (G.C.); riccardo.melloni@studio.unibo.it (R.M.); chiara.zanotti4@studio.unibo.it (C.Z.); giulio.desimone2@studio.unibo.it (G.d.S.); danila.pilastro@studio.unibo.it (D.P.); vittorio.lodi@unibo.it (V.L.); 2 Unit of Occupational Medicine, Sant’Orsola-Malpighi Hospital, 40138 Bologna, Italy; 3 Stony Brook Cancer Center, Stony Brook University, Stony Brook, NY 11794, USA

**Keywords:** COVID-19, vaccination, vaccine hesitancy, flu, health care workers

## Abstract

Background: While the uptake of the COVID-19 vaccine among healthcare workers (HCWs) is suboptimal, vaccine hesitancy has not been characterized in detail in this population. Objective: The aim of this study was to compare the prevalence of health-related conditions reported by HCWs during the COVID-19, 2020/21 flu, and 2019/20 flu vaccination campaigns, so to test the hypothesis that HCWs were more prone to report health conditions during the COVID-19 campaign. Methods: We analyzed vaccination questionnaires of 176 hospital-based HCWs who underwent the COVID-19 and the 2020/21 flu vaccinations; 2019/20 flu vaccination questionnaires were available for 130 of them. Outcomes included self-reported allergies, chronic diseases, and use of medications. We tested for prevalence equality, analyzed differences using the kappa statistics and concordance correlation, and explored factors associated with differences in reporting. Results: There was no difference in the proportion of HCWs reporting allergies in the three questionnaires, while chronic diseases were more frequently reported in the COVID-19 than in both 2020/21 (*p* = 0.04) and 2019/20 flu questionnaires (*p* = 0.02). Furthermore, a higher proportion of HCWs reported medications use in the COVID-19 vaccination questionnaire, compared to both the 2020/21 and the 2019/20 flu vaccination questionnaires (*p* < 0.001 for both). In each vaccine campaign, women reported more conditions than men, and the difference between chronic disease reports was greater for women than for men. Conclusions: Our results show more frequent reporting of health conditions during the COVID-19 than the flu vaccination campaigns, providing quantitative evidence of hesitancy of HCWs towards the COVID-19 vaccine.

## 1. Introduction

The COVID-19 pandemic has represented a great challenge for health systems. One of the specific aspects of the emergency has been the controversy about the measures to contain the spreading of SARS-CoV-2 infection and to reduce hospitalization and death from the disease. Despite vaccination was presented as the primary solution, its implementation has been hampered by doubts and skepticism. Among the first population groups offered the newly developed COVID-19 vaccines were healthcare workers (HCWs). HCWs represent a population with high knowledge and practice of vaccination and a high potential exposure to the infection [1].

Several studies reported that in general HCWs showed a positive attitude to the COVID-19 vaccination campaign [2]. Nevertheless, the relatively high rates of HCWs who refused to be vaccinated have raised concern on the difficulties in controlling the spread of the infection. Studies conducted in Italy showed a suboptimal rate of adherence to the recommended annual flu vaccination among HCWs due to skepticism around its effectiveness and perception of influenza not being a dangerous disease [3].

To address the relatively low uptake of the vaccine, some countries made it mandatory among HCWs [4]. In Italy, the vaccination campaign among HCWs started in December 2020 and lasted until late February–early March 2021, depending on the region. It could be hypothesized that the emergency situation related to the COVID-19 epidemic increased the adherence to the 2020/21 seasonal flu vaccination. In the general Italian population, the prevalence of vaccination was 23.7% in the 2020/21 flu campaign compared to 16.8% in the previous year [5]. As vaccination is a voluntary medical procedure requiring informed consent, subjects are commonly asked to fill a questionnaire assessing their health status. 

The aim of this study was to compare the attitude of a population of HCWs towards three different vaccinations campaigns, including the COVID-19 vaccination campaign and the 2020/21 and 2019/20 flu vaccination campaigns, by analyzing the questionnaires compiled on these three circumstances. Our aim was to test the hypothesis that HCWs reported more potentially adverse conditions during the COVID-19 campaign than during either flu vaccination campaign, reflecting some level of hesitancy towards the newly developed vaccine. A secondary hypothesis was that hesitancy toward the COVID-19 vaccine also impacted the 2020/21 flu campaign that took place during the COVID-19 pandemic, resulting in a higher proportion of HCWs reporting potentially adverse conditions in the 2020/21 flu campaign compared to the 2019/20 one.

## 2. Methods

The study was conducted on HCWs employed at the University Hospital of Bologna (Sant’Orsola-Malpighi Hospital). During the 2019/20 and 2020/21 flu vaccination campaigns, HCWs were vaccinated in the occupational health service within the hospital premises; for the COVID-19 campaign, they were invited to attend vaccination facilities set up outside the hospital, where other high-risk groups and eventually the general population were also vaccinated. The flu vaccines that were proposed were Fluarix Tetra, against 2A and 2B influenza strains, while the COVID-19 vaccine was Pfizer-BioNTech, consisting of 2 doses. The flu vaccination campaigns took place between October 2019 and January 2020 and between October 2020 and January 2021, respectively. The HCWs COVID-19 campaign started on 27 December 2020, and most of the subjects received the first dose by March. Overall, in the University Hospital of Bologna, 2780 HCWs were vaccinated against the flu in 2019/20, 5489 against the flu in 2020/21, and 4842 against COVID-19 in January 2021. In the flu vaccination campaigns, a standardized flu questionnaire was used at the time of vaccination to collect information on general health, different conditions, and medications use (Appendix A). A slightly expanded form was used in the COVID-19 campaign (Appendix B). The questionnaires were compiled by the HCWs before vaccine administration and checked by the occupational physician attending the vaccination. 

Inclusion criteria comprised employment at the University Hospital during the three vaccination campaigns and at the time of the study and having received the flu vaccinations at the Occupation Health Unit of the University Hospital and both doses of the COVID-19 vaccines at the largest vaccination site (Bologna-Fiera, Bologna, Italy). Potential participants were randomly selected from the roster of HCWs who participated in the 2020/21 flu vaccination campaign: the roster also included the personal phone number. Potential participants were contacted by telephone to confirm eligibility and obtain informed consent to participate in the research. Up to three phone calls were made in order to reach subjects to be enrolled.

**Figure 1:**
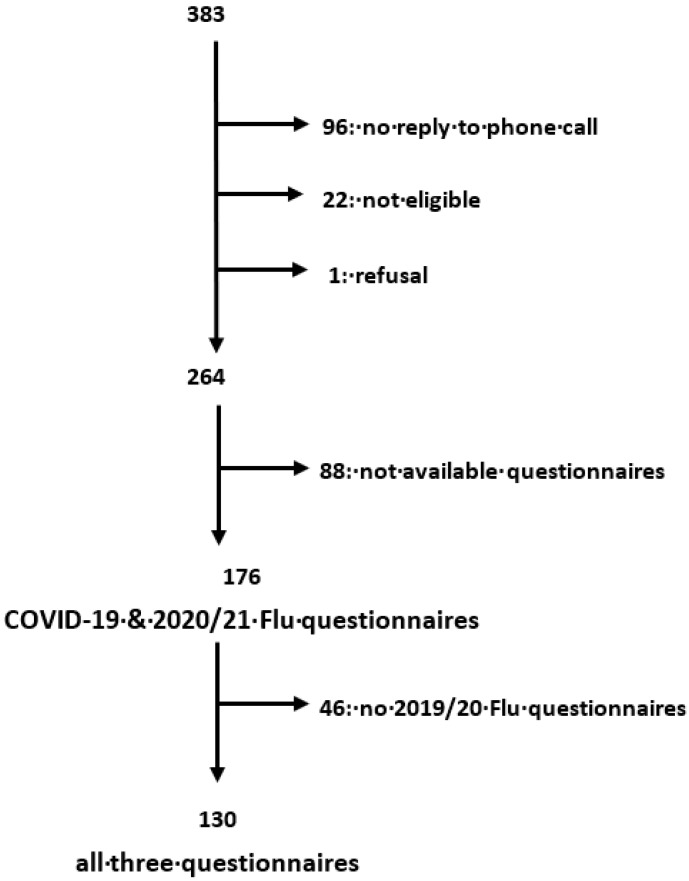
Selection process of the study population.

We identified 383 potential participants. Among them, 96 did not reply to the phone calls, 22 were not eligible, and 1 refused to participate in the research. We therefore included a total of 264 eligible and consenting HCWs. The COVID-19 and 2020/21 flu questionnaires were filled out by 176 of them, and the 2019/20 flu questionnaire was filled out by 130. Figure 1 illustrates the process of selection of the study population. 

We abstracted the questionnaires compiled during the three vaccination campaigns (2019/20 flu, 2020/21 flu, and first-dose COVID-19) according to a standardized form. 

We considered three outcomes: reported prevalence of allergies (whether any type of allergy was reported on each of the three questionnaires), reported prevalence of any chronic disease (whether any chronic disease was reported on each of the questionnaires), and reported chronic use of medications/supplements (whether the use of any medication or supplement was reported). 

We distinguished four types of allergies based on the information reported by the participants, i.e., antibiotics, non-steroidal anti-inflammatory drugs (NSAIDs), other drugs, and antigens other than drugs or unknown. If a subject reported multiple allergies, we considered only one, with a priority order (antibiotics > NSAIDs > other drugs > other/unspecified). 

The study was approved by the Institutional Review Board of the University of Bologna (n. 61143 del 15/03/2021).

### Statistical Analysis

First, we analyzed the distribution of the outcomes and compared them between the three questionnaires. We also analyzed their determinants in multivariate logistic regression models adjusted for sex, age category, and job title.

Next, we computed the kappa statistics to assess the agreement between questionnaires to assess whether the observed data significantly deviated from perfect concordance and tested whether the proportion of positive answers followed the pattern COVID-19 > 2020/21 flu > 2019/20 flu, using the concordance correlation coefficient (CCC) [6]; we also conducted multiple multivariate logistic regressions to investigate the potential determinants of discordance between questionnaires, using concordant answers as the reference category.

We used the commands kap, concord, prtest, logistic, and mlogit of Stata v. 16 (Statacorp, College Station, TX, USA).

## 3. Results

We included a total of 176 HCWs in the analysis of the COVID-19 and 2020/21 flu questionnaires, 130 of whom were included in the analysis comprising also the 2019/20 flu questionnaires. These subjects received the first dose of the SARS-CoV-2 vaccine in January 2021. A history of positive COVID-19 test was reported by 8/171 HCWs (4.7%; this information was missing for 5 subjects). 

Table 1 shows selected characteristics of the study population. The majority of HCWs included in the study were women. The mean age reported in the COVID-19 questionnaire was 42.6 years (SD, 11.5 years); medical doctors represented the main occupational group.

The distribution of the questionnaire data is reported in Table 2. Supplementary Table S1 shows in detail the specific conditions reported by the study subjects. 

There was no difference in the proportion of HCWs reporting allergies on the three questionnaires. The most commonly reported allergy on each of the questionnaires was to allergens other than drugs, but the proportion was lower for the COVID-19 questionnaire than for either flu questionnaire (9.2%, 18.2%, and 20.0% for the COVID-19, 2020/21 flu, and 2019/20 flu questionnaires, respectively). Conversely, the proportion of HCWs reporting an allergy to antibiotics was higher for the COVID-19 questionnaire (8%, 4%, and 1.5% for the COVID-19, 2020/21 flu, and 2019/20 flu questionnaires, respectively), but the difference was significant only with respect to the 2019/20 flu questionnaire. Women and subjects aged 36–50 years reported more often allergies in each of the campaigns. 

The number of HCWs reporting chronic diseases was higher in the COVID-19 questionnaire than in both the 2020/21 flu questionnaire (*p* = 0.04) and the 2019 flu questionnaire (*p* = 0.02), while no difference was observed between the two flu questionnaires (*p* = 0.25). During the COVID-19 and the 2020/21 flu campaigns, chronic diseases were more often reported by women. 

The proportion of HCWs reporting chronic use of medications or supplements was comparable for the two flu questionnaires (14.6% for the 2019/20 flu questionnaire and 18.2% for the 2020/21), while it increased to 46.0% for the COVID-19 questionnaire (*p* < 0.0001 for the hypotheses COVID-19 > each flu questionnaire). A higher proportion of women reported medications or supplements use, with more than half of them declaring any use in the COVID-19 vaccination (56.4%) questionnaire compared to 12.2% in the flu 2019/20 questionnaire. 

Out of the 176 HCWs included, 16 reported no PCR tests for SARS-CoV-2 infection in the previous month (9.1%), while 160 reported at least one recent test (90.9%); specifically, 151 (85.8%) HCWs declared one, 7 (4.0%) declared two, and 2 (1.1%) declared three recent tests. Information on contacts with subjects infected with SARS-CoV-2 in the last month was available for 172 HCWs; 92 of them (53.5%) declared none, while 49 (28.5%) reported at least one contact, and 31 (18.0%) answered that they did not know (not shown in detail). 

The proportion of HCWs reporting immunodeficiency was low and showed no difference among the three groups. Almost all participants reported no symptoms possibly related to COVID-19 infection when asked in the COVID-19 vaccination questionnaire. 

In the analysis of determinants of the three primary outcomes (allergies, chronic diseases, and medications/supplements use) declared in the COVID-19 questionnaire, no factors were associated with allergies. HCWs aged 51–67 years were more likely to declare chronic diseases (OR = 4.10, 95% CI = 1.03–16.3) compared to the younger group. Women declared medications/supplements use more often than men (OR = 4.41, 95% CI = 2.00–9.72).

In univariate analysis, the association between job category and each outcome showed that health assistants were more likely to report chronic diseases compared to physicians in the COVID-19 (OR = 3.33, 95% CI = 1.20–9.23) and the 2020/21 flu questionnaires (OR = 3.60, 95% CI = 1.18–11); the same association was found for medications/supplements use in the two flu campaigns (OR = 4.13, 95% CI = 1.37–12.5 in 2020/21 and OR = 4.93, 95% CI = 1.22–20.0 in 2019/20). In general, physicians tended to report non-significantly less conditions than participants in the remaining job categories. 

Despite a very high level of concordance between reports of primary outcomes at each vaccination (*p* of kappa and CCC < 0.01 for all comparisons between COVID-19 and 2020/21 flu, and between 2020/21 flu and 2019/20 flu, and for comparisons of allergies and medications/supplements use between COVID-19 and 2019/20 flu), a sizable number of subjects reported different answers on the three occasions. We therefore analyzed characteristics associated with discordant reporting via multivariate logistic regression. Table 3 shows the number of HCWs reporting the outcomes in each pairwise combination of questionnaires, and Table 4 reports the results of the corresponding multivariate analysis for the comparison between COVID-19 and 2020/21 flu questionnaires. The only significant factor associated with positive reporting in the COVID-19 questionnaire following a negative reporting in the flu 2020/21 questionnaire was female sex in the case of medications/supplements use (OR = 3.73; 95% CI = 1.76–7.90). 

The comparison of the 2020/21 and 2019/20 flu questionnaires showed none of the factors was significantly associated with discordant answers for the three outcomes (Supplementary Table S2). When comparing COVID-19 and 2019/20 flu questionnaires, no factor was associated with discordant answers for allergies and chronic diseases, whereas women were almost seven times more likely to declare medications/supplements use (*p* < 0.001), with all the HCWs who reported any use in 2019/20 also reporting it in the COVID-19 vaccine occasion (Supplementary Table S3). 

## 4. Discussion

The results confirmed our hypothesis of a higher proportion of HCWs reporting chronic diseases and use of medications or supplements in occasion of the COVID-19 vaccination campaign compared to both the 2020/21 and the 2019/20 flu vaccination campaigns, which may be interpreted as a marker of hesitancy toward the newly developed vaccine. No difference was observed for reports of allergies. Women were more likely to report conditions, and physicians were less likely to report them. Most of the available studies reported results based on surveys investigating hesitancy before COVID-19 vaccination uptake [7,8,9,10,11,12,13], while our analysis is based on multiple real-time surveys obtained through the forms compiled on the very day of the vaccination. 

When looking at the types of allergies, the number of drug-related allergies increased according to the trend flu 2019/20 < flu 2020/21 < COVID-19 campaign (Table 2). This may suggest higher accuracy in reporting this condition, but it can also be an indicator of a higher alert towards the safety of the COVID-19 vaccine and the fear for serious adverse events (i.e., anaphylactic shock) and, in the end, be a sign of hesitancy as allergies constituted the main and only element condition requiring precautions, especially in the first weeks of COVID-19 vaccine administration. 

While the proportion of HCWs reporting medications/supplements use was the highest for the COVID-19 questionnaire, it was higher for the 2020/21 flu questionnaire compared to the 2019/20 one, when the same form was used. Possible reasons for these results might be the fear for adverse events related to the concomitant use of drugs and an overall concern about the safety of vaccines caused by the development of the novel COVID-19 vaccine. Moreover, when only considering therapies for chronic diseases (e.g., antidiabetics, anti-hypertensives, beta-blockers, synthetic thyroid hormones, etc.), the rate was still higher for the COVID-19 than for the flu questionnaires. 

With concern to additional conditions such as malignancies, we were not able to use them as a marker of hesitancy because of their low prevalence. Despite this, it is reasonable to hypothesize that subjects would be more accurate in reporting cancer than other minor conditions such as those we selected as hesitancy markers.

Hesitancy toward COVID-19 vaccination is mainly related to fear of adverse events and perception of low vaccine effectiveness [13], which spread during the COVID-19 pandemic due to the fast development of the vaccines, the novelty of their formulation based on mRNA particles, and the relatively limited data on their safety. Besides this, flu vaccination also rose concern despite its long-time use, including among HCWs who are strongly recommended to adhere to annual vaccination campaigns. Indeed, HCWs’ attitude is usually influenced by the perception of not needing the vaccine given their usual good health, together with the belief that the flu vaccine may cause the disease by itself [3]. 

Our study confirmed that HCWs manifested markers of hesitancy at the time of COVID-19 vaccine administration, which in part influenced also the 2020/21 flu vaccination campaign that took place in the midst of COVID-19 vaccine development and of controversies about its safety. The number of HCWs of the University Hospital of Bologna who were vaccinated almost doubled from 2019/20 to 2020/21, and the proportion of those vaccinated against COVID-19 was very high already in the month of January 2021. Indeed, the higher proportion of HCWs who were vaccinated in the first anti-COVID-19 campaign indicates the effectiveness of initiatives to raise consciousness about the high risks for health posed by the COVID-19 emergency, as well as of the strong recommendation for COVID-19 vaccination, which was not compulsory at that time. Along with concern for safety and efficacy of the COVID-19 vaccines due to their novelty, a possible reason for the higher hesitancy toward BNT162b2 mRNA COVID-19 vaccine compared to the flu vaccine is the fact that the former requires two doses. Dror et al. described a higher rate of hesitancy towards COVID-19 vaccine compared to flu vaccine among HCWs [7]. 

The healthcare setting represents a high-risk environment for virus transmission. Consequently, it is mandatory for Italian HCWs to be fully vaccinated against several agents, including hepatitis B virus, measles, and diphtheria–tetanus–pertussis (DTP). In addition, HCWs are annually offered flu vaccination, which can be compulsory based on local policies. Despite this, the flu coverage in the hospital setting remains suboptimal. The COVID-19 pandemic urged public health authorities to update vaccination advice and regulations, in particular those addressing hospital settings and personnel. In summer 2020, the rate of vaccinated people to obtain herd immunity for COVID-19 was thought to be between 55% and 82%, and the rate of people willing to be soon vaccinated was estimated to be only 30% [13]. More recent studies indicate 70% as the threshold for heard immunity [14]. Different surveys collected data on the intention to receive the COVID-19 vaccine, with refusal rates between 10% and 23% among HCWs and students [9,10,11]. Of the 1546 participants of a study conducted among Qatari HCWs, 61% reported to receive flu vaccine annually, and more than 60% referred chronic conditions, though only 12% of those in the hesitant group reported any. In this study, female sex and concern on safety and effectiveness of the vaccine were predictors of hesitancy, while chronic conditions were not [10]. A survey of 13,462 people from 19 countries reported high rates of willingness to be administered the COVID-19 vaccine, with 46.8% of the participants completely agreeing and 24.7% of them somewhat agreeing overall; in this survey, Italy registered 70.8% of positive responses [12]. 

The United States represent a reference for the effectiveness of the introduction of compulsory flu vaccination in hospital settings, which was first introduced in Seattle in 2005 [1]. This led to a vaccination uptake in more than 98% of 5000 workers within the following four vaccination campaigns [15]. One interesting intervention that has been implemented in order to increase the uptake of flu vaccination was requiring the use of surgical masks during the flu season among unvaccinated HCWs in 2013 in New York, which turned out to be effective [16]. The low rate of flu coverage in hospital settings, despite a deeper knowledge about health and disease and a greater exposure to infection, suggests that even a well-informed and high-sensitized population can be affected by skepticism and distrust toward immunization programs [17]. 

In order to enhance vaccines uptake, a prolonged timeframe of vaccination campaigns, free access, and advertisement of vaccinations could be implemented both in the hospital setting and in the public sector. 

To date, few studies addressed the vaccination rate in the last flu seasons among HCWs. Moreover, most of the available studies focused on the attitude towards COVID-19 vaccination, limiting the observation to the period before vaccine administration. Our study represents the first attempt to investigate the attitude of HCWs towards the new COVID-19 vaccine based on data obtained at the time of vaccine administration. It is also the first study that compared the attitudes towards COVID-19 and earlier flu vaccinations, enlightening differences in reporting health conditions during each vaccination campaign.

Our study analyzed data obtained at the very moment of vaccine administration rather than before vaccination. The results of the comparison between the 2020/21 flu and the COVID-19 vaccines are therefore particularly valuable, as the two questionnaires were administered within a short time interval from each other, reducing the likelihood that any potential difference would correspond to real changes in the subjects’ health profiles. In addition, the results refer to the same sample of HCWs, thus avoiding the possibility that differences were caused by the different characteristics of the subjects receiving each vaccine. Additionally, we enrolled subjects administered the COVID-19 vaccine in January 2021, very close to the period of the 2020/21 flu campaign, providing high sensitivity to detect any difference between the two campaigns. For the same reason, we focused on the first COVID-19 vaccine dose. 

This study has some limitations. First, a selection bias could have been introduced during the enrolment. A possible reason is that one of the inclusion criteria was having received a previous flu vaccination during at least the 2020/21 campaign, thus excluding those less used to accept recommended vaccines. Indeed, the history of past flu vaccination has been associated to lower hesitancy [1]. If this bias occurred, it was therefore likely to produce an underestimate of the level of vaccine hesitancy. Another limit is the smaller number of flu 2019/20 questionnaires available, which may reflect the lower proportion of HCWs who were administered the flu vaccine in 2019/20. In addition, the COVID-19 and the flu questionnaires were slightly different and were administered in different settings, possibly influencing the subjects’ reporting. Despite this, we could compare multiple pieces of information collected at the time of vaccination. Furthermore, differences between the 2020/21 and the 2019/20 flu questionnaires, that were based on the same form, are consistent with those detected with the COVID-19 questionnaire, thus suggesting real differences rather than an artifact due to the forms’ structure. 

## 5. Conclusions

In conclusion, we found differences in health-related conditions reported in questionnaires for COVID-19 vaccination compared to those for the two previous flu campaigns, particularly concerning chronic conditions and medications/supplements use, with a higher proportion of reported conditions and larger differences between questionnaires for women. HCWs need to be sensitized to the recommended vaccinations, especially during the COVID-19 epidemic. Further studies with real-time collection of information and perspective design are needed to better understand this urgent topic.

## Figures and Tables

**Table 1:** Selected characteristics of the study population.

Characteristic	COVID-19 and 2020 Flu Questionnaires (*N* = 176)	All Three Questionnaires (*N* = 130)
Sex Male Female	66 (37.5%) 110 (62.5%)	56 (43.1%) 74 (56.9%)
Age 24–35 36–50 51–67	60 (34.1%) 63 (35.8%) 53 (30.1%)	48 (36.9%) 43 (33.1%) 39 (30.0%)
Job Medical doctor Nurse Healthcare assistant Medical technician	60 (34.1%) 54 (30.7%) 35 (19.9%) 27 (15.3%)	50 (38.5%) 41 (31.5%) 20 (15.4%) 19 (14.6%)

**Table 2:** Selected conditions reported on questionnaires.

Condition	COVID-19 (*N* = 176)	2020 Flu (*N* = 176)	2019 Flu (*N* = 130)
Do you feel well today? No Yes I do not know	0 (0%) 174 (98.7%) 2 (1.14%)	1 (0.6%) 174 (99.4%) NA	0 (0%) 130 (100%) NA
Allergies † No Yes	133 (76.4%) 41 (23.6%)	129 (73.3%) 47 (26.7%)	99 (76.1%) 31 (23.9%)
Type of allergy Other than drugs/unspecified Antimicrobial drugs NSAIDs Other drugs	16 (9.2%) 14 (8.0%) 6 (3.4%) 5 (2.9%)	32 (18.2%) ** 7 (4.0%) 4 (2.3%) 4 (2.3%)	26 (20.0%) ** 2 (1.5%) ** 0 (0%) 3 (2.3%)
Adverse event from vaccinations No Yes I do not know	171 (89.3%) 1 (0.57%) 2 (1.15%)	174 (98.6%) 2 (1.14%) NA	128 (98.5%) 2 (1.54%) NA
Vaccinations in the previous 4 weeks No Yes	163 (93.7%) 11 (6.3%)	175 (99.4%) ** 1 (0.6%)	126 (96.9%) 4 (3.1%)
Neurologic diseases No Yes	166 (95.4%) 8 (4.6%)	170 (96.6%) 6 (3.4%)	122 (93.8%) 8 (6.2%)
Chronic diseases † No Yes	134 (76.6%) 41 (23.4%)	147 (83.5%) * 29 (16.5%)	112 (86.1%) ** 18 (13.9%)
Malignant diseases No Yes	171 (97.2%) 5 (2.8%)	173 (98.3%) 3 (1.7%)	128 (98.5%) 2 (1.5%)
Chronic medications or supplement use No Yes †^ Oral contraceptive use Supplements Drugs acting on the central nervous system Drugs for chronic diseases Anti-cancer drugs	95 (54.0%) 81 (46.0%) 19 (10.8%) 27 (15.4%) 15 (8.6%) 41 (23.4%) 1 (0.6%)	144 (81.8%) ** 32 (18.2%) NA	111 (85.4%) 19 (14.6%) NA

Numbers may not add up to totals because of missing values. † Primary outcome. ^ 19 (10.8%) subjects reported the use of multiple categories of medications. Comparison with the COVID-19 questionnaire: * 0.10 > *p* > 0.05; ** *p* < 0.05. NA = Not available; NSAIDs = Non-steroidal anti-inflammatory drugs.

**Table 3:** Number of outcomes reported in each pairwise combination of questionnaires.

Comparison COVID-19–2020 Flu			Comparison COVID-19–2019 Flu			Comparison 2020 Flu–2019 Flu		
	Flu 2020			Flu 2019			Flu 2019	
	No	Yes		No	Yes		No	Yes
Allergies								
COVID 19 No	121 (94.5%)	12 (26.1%)	COVID-19 No	90 (90.9%)	12 (38.7%)	Flu 2020 No	93 (93.3%)	8 (25.8%)
Yes	7 (5.5%)	34 (73.9%)	Yes	9 (9.1%)	19 (61.3%)	Yes	6 (6.1%)	23 (74.2%)
Chronic diseases								
COVID 19 No	129 (88.4%)	5 (17.2%)	COVID-19 No	97 (86.6%)	3 (16.7%)	Flu 2020 No	107 (95.5%)	3 (16.7%)
Yes	17 (11.6%)	24 (82.8%)	Yes	15 (13.4%)	15 (83.3%)	Yes	5 (4.5%)	15 (83.3%)
Medications/ supplements								
COVID 19 No	92 (63.9%)	3 (9.4%)	COVID-19 No	73 (65.8%)	0 (0.0%)	Flu 2020 No	102 (91.9%)	4 (21.1%)
Yes	52 (36.1%)	29 (90.6%)	Yes	38 (34.2%)	19 (100%)	Yes	9 (8.1%)	15 (78.9%)

**Table 4:** Odds ratios for discordance in reporting the outcomes (positive or negative answer to questions about allergies, chronic diseases, and medications/supplements use) between COVID-19 and 2020 flu questionnaires (reference category: concordant reports).

Characteristics	Positive at COVID-19, Negative at 2020 Flu OR, 95% CI	Negative at COVID-19, Positive at 2020 Flu OR, 95% CI
**Allergies**		
Age (years) 24–35 36–50 51–67	Ref 3.57, 1.03–12.3 1.66, 0.36–7.58	Ref 3.86, 1.23–12.1 4.45, 1.33–14.8
Sex Male Female	Ref 1.96, 0.67–5.72	Ref 4.30, 1.60–11.6
Job Medical doctor Nurse Healthcare assistant Medical technician	Ref 1.22, 0.37–4.05 0.29, 0.05–1.64 0.47, 0.08–2.66	Ref 1.33, 0.46–3.87 0.52, 0.15–1.82 0.56, 0.14–2.26
**Chronic diseases**		
Age (years) 24–35 36–50 51–67	Ref 1.40, 0.34–5.76 1.94, 0.45–8.40	Ref 0.55, 0.01–1.32 NA
Sex Male Female	Ref 0.65, 0.23-1.80	Ref 0.95, 0.13-7.01
Job Medical doctor Nurse Healthcare assistant Medical technician	Ref 0.85, 0.21–3.41 1.09, 0.23–5.23 1.03, 0.21–5.09	Ref NA 13.5, 0.81–225.8 NA
**Use of medications or supplements**		
Age (years) 24–35 36–50 51–67	Ref 0.92, 0.39–2.19 1.30, 0.51–3.33	Ref 0.80, 0.04–17.4 1.03, 0.04–27.8
Sex Male Female	Ref 4.25, 1.89–9.56	Ref 1.62, 0.14–18.9
Job Medical doctor Nurse Healthcare assistant Medical technician	Ref 0.91, 0.37–2.22 0.74, 0.25–2.17 0.90, 0.30–2.66	Ref 1.11, 0.05–22.8 1.65, 0.06–46.3 NA

OR, odds ratio, adjusted for sex, age, and job category. CI, confidence interval. Ref, reference category.

## Data Availability

Data available to bona-fide investigators upon request.

## Supplementary Materials

The following are available online at https://www.mdpi.com/article/10.3390/vaccines9111312/s1, Table S1: List of conditions ever specified by the participants in the three questionnaires. Table S2: Odds ratios for discordance in reporting between 2020 flu and 2019 flu questionnaires (reference category: concordant reports); Table S3: Odds ratios for discordance in reporting between COVID-19 and 2019 flu questionnaires (reference category: concordant reports).

## Appendix A. Flu Questionnaire (Translated from the Italian Version)

1. Are you feeling well today?
2. Did you ever suffer from convulsions, epilepsy, or other neurological diseases?
3. Are you affected by any allergy to medications, food, latex, or other substances?
4. Did you ever have severe adverse events after vaccination?
5. Do you suffer from immune system diseases or other severe diseases such as cancer, leukemia, HIV?
6. Do you suffer from chronic diseases of immune, rheumatic, metabolic, cardiac, respiratory, renal nature? Do you suffer from chronic diseases affecting other organs? Do you suffer from coagulation-related disorders?
7. In the last 6 months, have you undergone long-duration therapies? Have you taken any medications on a regular basis? Have you undergone dialysis or radiotherapy?
8. Have you ever undergone surgical operations?
9. Did you take any vaccination shot in the last 4 weeks?
10. If woman, are you pregnant or is there the possibility you will be pregnant in the next month?

Additional notes:

## Appendix B. COVID-19 Questionnaire (Translated from the Italian Version)

Are you ill at the moment?

1. Do you have a fever?
2. Are you affected by any allergy to latex, food, medications, or any of the vaccine’s components?
3. Did you ever have severe adverse event after vaccination?
4. Do you suffer from cardiac, pulmonary, renal diseases? Do you suffer from asthma? Do you suffer from diabetes? Do you suffer from anemia or other blood-related diseases?
5. Do you suffer from a condition which is compromising your immune system (cancer, leukemia, lymphoma, HIV/AIDS, organ transplantation)?
6. In the last 3 months, did you take medications which can make your immune system weaker (example: steroids)? Did you take antitumoral medications? Did you undergo radiation therapy?
7. In the last year, did you receive any blood or blood products transfusion, and did you take any immunoglobulins or antiviral medications?
8. Did you ever suffer from convulsions or any disease affecting your brain or nervous system?
9. Did you take any vaccination shot in the last 4 weeks?
10. Do you use anticoagulants?

Specify in the following lines the medications, in particular, anticoagulants, supplements, vitamins, minerals, or other possible alternative medicaments you are currently assuming:

FOR WOMEN:

Are you pregnant, or are you planning to get pregnant in the next month?

Are you breastfeeding?

COVID-RELATED ANAMNESIS

In the last month have you been in contact with a person affected by SARS-CoV-2?

Do you have any of the following symptoms?

-Cough/cold/fever/dyspnea/flu-like symptoms

-Sore throat/ loss of taste/loss of smell

-Abdominal pain/diarrhea

-Abnormal bruises, bleedings/red eyes

Did you do any international trips in the last month?

COVID-19 test:

-No recent test

-In possess of the result of a recent COVID-19 test

COVID-19 Negative test (Date: ......................................)

COVID-19 Positive test (Date: ........................................)

-Waiting for the result of a recent COVID-19 test (Date: ...................................)

Please report other possible diseases or useful information on your health status.
