# Supplementary material for: Comparing the Attitude toward the COVID-19 and the 2020/21 and 2019/20 Flu Vaccination Campaigns among Italian Healthcare Workers
Source: Vaccines (Basel). 2021 Nov 11;9(11):1312. doi: 10.3390/vaccines9111312 (PMC8618503; doi:10.3390/vaccines9111312)
Supplement: Supplementary file 1 [file vaccines-09-01312-s001.zip › vaccines-1451875-supplementary.pdf]

vaccines

MDPI

Supplementary S1. List of conditions ever specified by the participants on the three questionnaires.

Allergies	
Other than Drugs/Unspecified	Mites, Animal Fur, Grasses, Pollen, Olive, Foods, Cosmetics, Nickel, Thimerosal
Antibiotics	Penicillin, Macrolides, Sulfonamides
NSAIDs	NA
Other Drugs	Steroids, Paracetamol, Muscle Relaxant, Anesthetics
Chronic diseases	Hypothyroidism/thyroiditis, type 2 diabetes, osteoporosis, hypertension, myocardial infarction, congenital tachycardia, hypercholesterolaemia, heart valve disease, cardiopathy, aortic aneurism, colitis, UC, GERD, CD, megacolon, HCV, anxiety/depression, fibromyalgia, psoriasis, hemicrania, abdominal adherence, essential thrombocythemia, renal dysplasia, MEN2A mutation, chorioretinopathy, polyneuropathy, Charcot–Marie–Tooth polyneuropathy, MS, angioedema, narrowing of the vertebral canal, osteochondritis
Malignancies	Breast cancer, colorectal cancer, thyroid cancer, Kaposi sarcoma, dermatofibrosarcoma
Surgery	Caesarian section, appendicectomy, tonsillectomy, gastrectomy, mastectomy, thyroidectomy, aortic aneurism, nevus removal, ovarian cysts removal, gynecological surgery, eyes surgery, liposuction, aesthetic surgery
Previous vaccinations	DTP, HPV, flu
Adverse reaction to vaccinations	Urticarial rashes to measles vaccination; serious adverse event to pertussis vaccination in early childhood
Any possible notes	Allergies, chronic diseases, malignancies, use of medications, surgery, head injury, reaction to previous vaccines, history of COVID–19 infection, specification of first time to be vaccinated (for flu)

NA= not applicable; UC= ulcerative colitis; GERD= gastroesophageal reflux disease; CD= celiac disease; MS=Multiple Sclerosis; DTP= Diphteria–Tetanus–Pertussis.

Supplementary S2. Odds ratios for discordance in reporting between 2020 flu and 2019 flu questionnaires (reference category: concordant reports).

Characteristics	Positive at 2020 flu, Negative at 2019 Flu OR, 95% CI	Negative at 2020 Flu, Positive at 2019 Flu OR, 95% CI
	**Allergies**	
Age (years)		
24–35	Ref	Ref
36–50	NA	0.52, 0.07–3.96
51–67	NA	0.24, 0.02–3.52
Sex		
Male	Ref	Ref
Female	1.68, 0.28–10.1	5.15, 0.60–44.3
Job		
Medical doctor	Ref	Ref
Nurse	0.60, 0.07–4.90	0.33, 0.03–3.27
Healthcare assistant	0.59, 0.05–7.61	2.08, 0.22–19.8
Medical technician	0.58, 0.04–7.47	NA
	**Chronic diseases**	
Age (years)		
24–35	Ref	Ref
36–50	NA	1.16, 0.01–5.02
51–67	NA	0.12, 0.01–4.17
Sex		
Male	Ref	Ref
Female	NA	NA
Job		
Medical doctor	Ref	Ref
Nurse	0.75, 0.04–14.6	NA

Healthcare assistant	4.00, 0.25–63.9	NA
Medical technician	2.00, 0.09–44.5	NA
	**Use of medications or supplements**	
Age (years)		
24–35	Ref	Ref
36–50	0.44, 0.05–3.63	3.10, 0.22–43.7
51–67	0.96, 0.13–6.97	1.78, 0.08–41.2
Sex		
Male	Ref	Ref
Female	2.93, 0.55–15.7	0.75, 0.10–5.71
Job		
Medical doctor	Ref	Ref
Nurse	2.29, 0.31–16.7	0.45, 0.03–5.95
Healthcare assistant	7.50, 0.82–68.6	1.02, 0.07–15.7
Medical technician	NA	NA

OR, odds ratio, adjusted for sex, age and job category. CI, confidence interval. Ref, reference category. NA, not applicable.

Supplementary S3. Odds ratios for discordance in reporting between COVID–19 and 2019 flu questionnaires (reference category: concordant reports).

**Characteristics**	**Positive at COVID–19, Negative at 2019 Flu OR, 95% CI**	**Negative at COVID–19, Positive at 2019 Flu OR, 95% CI**
	**Allergies**	
Age (years)		
24–35	Ref	Ref
36–50	NA	1.17, 0.28–4.90
51–67	NA	0.22, 0.02–2.27
Sex		
Male	Ref	Ref
Female	2.02, 0.44–9.36	2.27, 0.57–9.03
Job		
Medical doctor	Ref	Ref
Nurse	4.58, 0.48–43.42.31, 1.80–29.6	0.85, 0.18–3.95
Healthcare assistant	NA	1.42, ʻ.20–10.1
Medical technician		0.05–5.44
	**Chronic diseases**	
Age (years)		
24–35	Ref	Ref
36–50	3.52, 0.78–15.9	NA
51–67	1.89, 0.34–10.7	NA
Sex		
Male	Ref	Ref
Female	2.37, 0.70–8.04	1.40, 0.11–17.3
Job		
Medical doctor	Ref	Ref
Nurse	0.91, 0.22–3.74	NA
Healthcare assistant	1.00, 0.18–5.45	NA
Medical technician	0.72, 0.11–4.59	NA
	**Use of medications or supplements***	
Age (years)		
24–35	Ref	Ref
36–50	0.87, 0.30–2.50	NA
51–67	1.18, 0.38–3.70	NA
Sex		
Male	Ref	Ref
Female	6.69, 2.51–17.8	NA

Job		
Medical doctor	Ref	Ref
Nurse	1.53, 0.54–4.36	NA
Healthcare assistant	1.56, 0.41–5.96	NA
Medical technician	1.13, 0.29–4.47	NA

OR, odds ratio, adjusted for sex, age and job category. CI, confidence interval. Ref, reference category. NA, not applicable.
* For medications or supplements use, no subject reported positive answer in the 2019 flu questionnaire without reporting it in the COVID–19 one. .
